# Computational Simulation of the Bioactivity of Selected Compounds Derived From the *Sapium* and *Salvias* Genera as Anticancer Agents

**DOI:** 10.1155/bmri/4962249

**Published:** 2026-03-14

**Authors:** Russell B. O. Ouma, Usama Raza, Silas M. Ngari, Joshua K. Kibet, Uzair Nisar, Muhammad Waqar Ali

**Affiliations:** ^1^ Department of Chemistry, Egerton University, Njoro, Kenya, egerton.ac.ke; ^2^ Department of Pharmacognosy, Dow University of Health Sciences, Karachi, Pakistan, duhs.edu.pk; ^3^ Department of Pharmacology, Ziauddin University, Karachi, Pakistan, zu.edu.pk; ^4^ Federal University of Arts Science and Technology, Karachi, Pakistan

**Keywords:** APE1 inhibition, enzyme inhibition, molecular docking, natural polyphenols

## Abstract

The investigation into the anticancer properties of plant extracts, which are known to be effective therapeutic agents with minimal side effects, is gaining significant traction. The current study examined the bioactive compounds extracted from *Sapium ellipticum* and *Salvia miltiorrhiza* Bunge. Salvianolic acid (Sal B), ellagic acid (EA), [20‐^3^H]‐12‐deoxyphorbol‐13‐isobutyrate ([^3^H] DPB), and [20‐^3^H] phorbol‐12, 13‐dibutyrate ([^3^H] PDBu) have all been analyzed in vitro and in silico. Based on the molecular docking simulations, Sal B targeted DNA lyase, topoisomerase II alpha, and mTOR, with docking scores of −9.5, −6.9, and −7.9 kcal/mol, respectively. The docking scores of these compounds were comparable with those of EA, which had a docking score of −8.7, −6.5, and −7.6 kcal/mol when targeting DNA lyase, topoisomerase II alpha, and mTOR, respectively. Remarkably, Sal B (−9.0 kcal/mol), EA (−7.6 kcal/mol), [3H] PDBu (−8.7 kcal/mol), and [3H] DPB (−7.1 kcal/mol) showed weaker binding affinities to the mTOR kinase enzyme compared with rapamycin (−11.2 kcal/mol). With an IC_50_ of 11.6 *μ*M, Sal B demonstrated remarkable efficacy and was almost as effective as the common inhibitor rapamycin (IC_50_ of 5.2 *μ*M). With an IC_50_ of 21.9 *μ*M, EA demonstrated a mild but similar inhibition. The assay and docking results were validated by the root mean square deviation (RMSD) plots of Sal B and EA, which demonstrated that they are both kinetically stable. The results of this study present a promising pathway for the advancement of therapeutic agents targeting breast cancer (BC) and prostate cancer (PCa). Eventually, these bioactive compounds will require precise clinical studies and in vivo testing to confirm their possible preventive and curative action.

## 1. Introduction

Early detection, as well as the development and application of new drugs, is pivotal in achieving desirable health outcomes for cancer patients [1]. Numerous controversies and questions exist in the management of breast cancer (BC) and prostate cancer (PCa), which directly impact daily practice. However, the present detection modalities and intervention strategies, including ultrasound, mammography, and magnetic resonance imaging (MRI), are associated with inherent limitations in specificity and sensitivity, further complicating timely and accurate diagnosis [2, 3]. It is well‐established in the literature that tumor cells lead to the disrupted expression of biomarkers, enzyme proteins, and nucleic acids, including microRNAs (miRNAs), androgen receptor (AR), and apurinic/apyrimidinic endonuclease 1 (APE1) [4–6]. Pharmacological inhibition of DNA repair holds great promise in targeting genetic differences between tumors and normal tissues. Recent scientific reports have also maintained that pharmacological inhibition of deoxyribonucleic acid (DNA) repair spares normal cells while exploiting genetic vulnerabilities of cancer cells [7, 8]. DNA base excision repair (BER) contributes to repairing alkylating‐damaged bases in DNA, including dacarbazine and temozolomide, as well as oxidative damage, spontaneous hydrolysis, and deamination [9]. DNA glycosylases are critical for BER, which is responsible for the identification and elimination of damaged bases, resulting in the generation of an apurinic/apyrimidinic site (AP site) [10]. The DNA backbone at the location of AP is then cleaved by APE1 via short‐patch or long‐patch BER cellular signaling [11, 12]. APE1 has been identified for other roles, including mitochondrial metabolism, regulation of biological functions that control redox homeostasis, and neovascularization. As such, APE1 is an attractive target in retinal ocular disease, inflammatory bowel disease (IBD), diabetic macular edema (DME), cancer, and chemotherapy‐induced peripheral neuropathy [13, 14].

Modern drug discovery approaches have leveraged molecular docking to predict how ligands (small molecules) interact with proteins, aiding in the identification of modulators or inhibitors of their function [15, 16]. Molecular docking algorithms are valuable tools for understanding the repurposing of existing drugs, discovering potential drug molecules, elucidating drug‐target interactions, and designing highly active enzymes [16, 17]. Molecular docking is fundamental to the current progress of accurate ligand–receptor modeling software and scoring functions. APE1 has been identified as a therapeutic agent for the suppression of breast and prostate tumor cells [13]. It is frequently found to be overexpressed, contributing significantly to tumor regression, resistance to therapy, and poor clinical outcomes [18, 19]. In BC, overexpression of APE1 is associated with DNA repair induced by reactive oxygen species, allowing tumor cells to proliferate under oxidative stress conditions that trigger apoptosis in normal cells. Drug resistance in anticancer medicines is a result of APE1′s enhanced DNA repair ability [20]. Additionally, APE1′s secondary function as a transcription factor (TF) redox regulator contributes to cancer cell survival, metastasis, and angiogenesis [18, 21].

Proteins such as mammalian target of rapamycin, also known as serine/threonine‐protein kinase (mTOR), and topoisomerase II alpha have also been identified by other research groups to be directly associated with the development and management of BC and PCa. According to research, topoisomerase II alpha is one of the key targets for chemotherapy drugs that inhibit the proliferation of cancer cells. The clinically approved topoisomerase I alpha target includes belotecan, topotecan, and camptothecin analog irinotecan [22, 23], whereas topoisomerase II alpha inhibitors comprise doxorubicin, pixantrone, etopophos, epirubicin, daunorubicin, ambrubicin, idarubicin, amsacrine, and mitoxantrone [24]. By reducing torsional tensions caused by overwound DNA during strand separation, topoisomerase II alpha plays significant roles in several physiological processes, including transcription, recombination, and DNA replication [24]. Due to their function in maintaining DNA topology during replication and transcription, both topoisomerase I alpha and topoisomerase II alpha (specifically the topoisomerase II alpha isoform) are implicated in the development of malignancy. Furthermore, serine/threonine protein kinases control a variety of biological processes, such as cell activity, proliferation, survival, and death. In recent years, this protein has gained considerable scholarly attention as a promising lead compound for treating and managing autoimmune disorders, viral diseases, inflammation, neurological diseases, and various types of cancer [24, 25]. Nevertheless, despite accumulating evidence from experimental and computational investigations, there is no universal “recipe” that facilitates the development of new protein inhibitors. Consequently, numerous research undertakings are geared towards developing new key inhibitors. Given their functions in DNA repair, cell division, and cancer survival, proteins are important targets in the detection, treatment, and prevention of cancer. Previous scientific studies have targeted enzymes involved in DNA repair, including mTOR [26–28], topoisomerase II alpha [29–31], and DNA lyase in cancer therapies.

*Sapium ellipticum* and other species in the same genus have been found to contain active compounds in extracts (alkaloids), which can serve as lead compounds during drug development [32]. Particularly, two important metabolites of *S. ellipticum*, [20‐^3^H] phorbol‐12, 13‐dibutyrate, abbreviated as [^3^H] PDBu, and [20‐^3^H]‐12‐deoxyphorbol‐13‐isobutyrate, abbreviated as [^3^H] DPB, have been shown to suppress inflammation and stop the proliferation of cancer cells. Both [^3^H] PDBu and [^3^H] DPB are capable of activating protein kinase C (PKC), which scientists have utilized to investigate cellular processes, including differentiation, immune responses, and growth [33–35]. EA is a promising chemical that could be extracted from *S. ellipticum* as a natural alternative in cancer treatment because other preclinical studies have shown that it possesses antimicrobial properties that neutralize free radicals and reduce DNA damage [36, 37]. Additionally, it inhibits DNA and topoisomerase II alpha, which suggests that it may have anticancer properties. It can also interfere with DNA processing enzymes like DNA lyase and control the production of APE1. Given its antioxidant qualities and capacity to interact with DNA, salvianolic acid B (Sal B) has also been shown in other research to be a promising anticancer agent. It is known to decrease DNA repair proteins such as x‐ray repair cross‐complementing (XRCC) protein and poly (adenosine diphosphate‐ribose) polymerase 1 (PARP1) and to prevent oxidative DNA damage. Sal B is a polyphenolic substance that was extracted from *Salvia miltiorrhiza* (commonly called Danshen) roots [38, 39]. Sal B has multiple hydroxyl groups, which enable it to form metal ion chelation and hydrogen bonding, thereby interfering with the magnesium–ion‐dependent catalytic activity of DNA lyases and enhancing apoptosis. Considering the widespread clinical use of Sal B in Asia and beyond, it is crucial to understand its mechanism to prevent complications and maximize its therapeutic benefits. However, the molecular characteristics of these natural products have not been thoroughly investigated, despite their importance as a reliable indicator of various kinds of chemical molecules, such as analgesics [40]. These characteristics are typically classified as physical, chemical, and biological [41]. Three computational techniques, including quantum mechanics, molecular dynamics, and molecular mechanics, can be used to calculate and determine these properties [42].

The current research is aimed at addressing this gap in knowledge by conducting a systematic investigation of the four ligands, EA, Sal B, [^3^H] DPB, and [^3^H] PDBu. They are promising lead compounds capable of interacting with serine/threonine‐protein kinase mTOR, APE1, and topoisomerase II alpha in combating cancer‐inducing agents. Accumulating academic output has also shown that the AR in PCa is linked to the enzyme topoisomerase II alpha, which is linked to the development of the tumor. Topoisomerase II alpha inhibitors are also utilized in BC chemotherapy. Additionally, oxidative DNA damage repair enzymes and DNA lyase, namely adenylosuccinate lyase (ADSL), have been implicated in the development of PCa and BC aggressiveness, respectively [43]. In addition, mTOR has been implicated in promoting PCa growth [44] and is being investigated as a therapeutic target in cancer care to overcome resistance [43]. This combined both theoretical and experimental study used molecular docking and in vitro assay to determine the docking scores and the inhibitory potential of the ligands in BC and PCa cell lines. The anticancer activity of all compounds explored in this study has been examined in vitro to validate the molecular dynamics and docking findings. The cell‐inhibitory effects of each ligand on the cell lines were also investigated to identify the bioactive compound with potential anticancer effects in targeting BC and PCa against standard drugs.

## 2. Materials and Methods

### 2.1. Molecular Docking of Ligands and Disease‐Causing Proteins

PubChem database was used to retrieve the ligands—Sal B (CID: 119177), EA (CID: 5281855), [^3^H] PDBu (CID: 3778, ^3^H] DPB (CID: 107855) and downloaded .sdf format processed to mol2 with Open Babel Version 3.1.1 and MMFF94 force field applied with Avogadro software Version 1.2.0 to optimize geometry [45]. The optimized energy values of the ligands were −183.2 kcal/mol for Sal B and −152.7 kcal/mol for EA. [3H] PDBu had an optimized energy value of −139.2 kcal/mol and [^3^H] DPB was −134.8 kcal/mol. AutoDock Tools Version 1.5.7 were used to convert the minimized structures to PDBQT format, followed by docking with AutoDock Vina Version 1.2.3. AutoDockVina was used to dock the ligand structures with the grid box position corresponding to the location of identified catalytic residues at PDB ID: 1DE8, APE1, DNA base excision protein kinase; PDB ID: 5GWK, serine/threonine protein kinase, and PDB ID: 4JSV, mTOR) [46–48] and subjected to preprocessing for docking. The PDB was accessed to retrieve the 3D structure of the target protein, downloaded in a .pdb file, and preprocessed [49]. AutoDock Tools v1.5.7 was used to prepare the protein, and crystallographic water molecules were eliminated, replaced by polar hydrogen, and Kollman charges were added. The nitrogen atoms were then protonated (made positive) and sp^3^‐hybridized, and the carboxyl groups were deprotonated (made negative). Depending on the parameters in the computational platform, this was done to create side chains, stabilize the charges, and saturate the missing residues [49]. The significant active site of a disease‐producing protein was predicted. Multiple active receptor sites may exist, but a single key pose was selected [49]. To enhance the scoring measurements heteroatoms and water molecules were removed. For accurate tautomeric configurations, the ligands were protonated at pH 7.4 before molecular docking. The grid box coordinates: (127.768545 (x), 28.633364 (y), and 109.181545 (z) were set with a center box of 15 Å for the DNA lyase ligand and for the mTOR protein; a grid setup with dimensions of −18.968439 (x), −32.923976 (y), and −56.432976 (z) was utilized. The grid was centered on the kinase′s adenosine triphosphate (ATP) binding domain, and the box size was 15 Å. The grid box coordinates of the catalytic amino acids and etoposides for the topoisomerase II alpha protein are 23.790037, −37.327815, and −55.582123 for x, y, and z dimensions, respectively. For all the docking calculations, the grid box coordinates were user‐driven, relying on structural biology data. Exhaustiveness was established at eight to achieve 10 poses for each ligand. The AutoDock Vina Version 1.2.3 performed the docking and generated the results, which included the docking score expressed as kilocalories per mole (kcal/mol) with the corresponding 2D and 3D atomic structures. Furthermore, we re‐docked the co‐crystallized ligands into their original binding pockets to determine the binding and validate the docking protocol [45, 49]. The low values of RMSD (less than 2.0 Å) that were obtained with the help of these controls are evidence that the binding sites were correctly defined. The interaction analysis was visualized using Discovery Studio software Version 21.1.0 to depict compound–protein interactions that involved pi–pi stacking, water‐mediated interactions, hydrophobic contacts, and hydrogen bonds [50, 51].

### 2.2. Calculation of Chemical Reactivity Descriptor

Density functional theory (DFT) was used to model the optimized geometries, energies, and cocrystal structures using Gaussian 09w with B3LYP (Becke three‐parameter Lee, Yang, and Parr) and the 3‐21G basis set. Several appropriate calculation formulas were employed to determine reactivity and chemical descriptors. The ionization potential, I, was calculated using the formula, I = −*ε* HOMO, energy gap *Δ*E_Gap_ from *Δ*E_Gap_ = *ε* HOMO— *ε* LUMO, and electron affinity, A, from A = −*ε* LUMO. Moreover, the electronegativity, *χ*, was calculated using the formula *χ* = (I + 1)/2, chemical potential, *μ*, from *μ* = −(I + 1)/2, hardness *η*, using the formula *η* = (I − A)/2, and electrophilicity, *ω*, was calculated from *ω* = *μ* ^2^/2*η*, whereas softness *σ* was obtained from *σ* = 1/*η*.

### 2.3. Molecular Dynamics Simulations

Molecular dynamics simulations were used to investigate the chemical stability during the protein complex′s development. Molecular dynamics simulations of the EA, Sal B, [^3^H] PDBu, and [^3^H] DPB protein complexes were conducted for up to 200 nanoseconds (ns) using GROMACS Version 2025.2 on Google Colab. Utilizing the AMBER99SB force field in the simulations allowed for a realistic representation of the binding position while also ensuring that docking stability was maintained [49]. The entire system was solvated in a triclinic water box, employing the transferable intermolecular potential with three points (TIP3P) water model [49]. After eliminating steric clashes through energy minimization using the steepest descent approach, equilibration phases under constant particle number, pressure, and temperature (NPT ensemble) and constant particle number, volume, and temperature (NVT ensemble) were conducted. The leapfrog integrator, in conjunction with the Berendsen thermostat and barostat algorithms, stabilized the system′s temperature and pressure at 300 K and 300 bar [49]. Accordingly, following Ouma et al.′s [49] method, the long‐range electrostatic forces were treated using the particle‐mesh Ewald approach and periodic boundary conditions. In molecular dynamics simulations, a cubic cell was transmitted through 20 Å. The MM/GBSA method was used to calculate the number of hydrogen bonds and the root mean square deviation (RMSD). PyMOL 2.5.2 and Igor Pro Version 5.04B were used for visualization.

#### 2.3.1. Analysis of In Vitro Enzyme Inhibition

Experiments were conducted in the Biosafety Level II laboratory at Ziauddin University, Karachi. Two human BC cell lines, MCF‐7 (RRID: CVCL_0031) and MDA‐MB‐231 (RRID: CVCL_0062), as well as two PCa cell lines, PC‐3 (RRID: CVCL_0035) and DU 145 (RRID: CVCL_0105), were used in this in vitro enzyme inhibition investigation. A male patient′s Grade IV PCa is represented by PC‐3, wheres a male patient′s prostate carcinoma with brain metastases is represented by DU 145 [49]. In vitro analysis was also performed on MCF‐7, a female luminal‐type adenocarcinoma, whereas MDA‐MB‐231, a female donor, triple‐negative subtype, was employed. These cell lines have been selected to mimic clinically relevant models of hormone‐responsive and hormone‐refractory cancers to test the phytochemicals [49]. The BC and PCa cell lines were supplied by the American Type Culture Collection (ATCC), a recognized and licensed supplier of authenticated biological material worldwide. Whereas PC‐3 and DU 145 were acquired in March 2024, MDA‐MB‐231 and MCF‐7 were acquired in January 2024 [49]. The Ziauddin University laboratory inventory management system was used to register and catalog each cell line that was received, which underwent analysis with a certificate of analysis and material safety data sheets (MSDS). To ensure traceability and standards, the following identities were supplied: MCF‐7 (ATCC HTB‐22), MDA‐MB‐231 (ATCC HTB‐26), PC‐3 (ATCC CRL‐1435), and DU 145 (ATCC HTB‐81). Frozen cell lines were cultivated in ATCC‐recommended media with 10% heat‐inactivated fetal bovine serum and 1% penicillin–streptomycin. A humidified carbon (IV) oxide (CO_2_) incubator set at 37°C was used to incubate the cultures. To maintain the integrity of the phenotype, the number of passages was limited to below 20. All four lines were authenticated using the Promega Power Plus 16 HS technology and short tandem repeat (STR) profiling. As previously reported in the assay, STR results revealed a genetic identity of approximately 95% with reference samples from the ATCC and Cellosaurus databases [52]. No signs of cell misidentification or cross‐contamination were found. Additionally, individual cell lines with previous allegations of contamination or misidentification were matched to the International Cell Line Authentication Committee (ICLAC) database and Cellosaurus. Since all cell lines were determined to be safe, they are extensively utilized as a benchmark in cancer research.

Using the MycoAlert PLUS Mycoplasma Detection Kit (Lonza), Mycoplasma contamination was tracked at baseline and at the conclusion of every 4 weeks [53]. Tests were conducted as per manufacturer protocols and provided negative or positive controls. Throughout the experiment, all cell lines displayed negative results for *Mycoplasma* contamination. These regular screenings formed essential quality control interventions, and only validated *Mycoplasma*‐free cultures would be used in experiment replicates. The work on cell culture was carried out only under the safety laboratory of Ziauddin University, and in accordance with local biosafety instructions and international biomedical research requirements [45, 49]. This strict adherence guaranteed the reproducibility and validity of cellular responses when scaled to the anticancer compound screening context.

#### 2.3.2. Compound Procurement and Lab Facility

Anticancer compounds were pharmacologically assessed in controlled labs through standardized in vitro assays [54, 55]. Sal B (Cat. No. HY‐N0210), EA (HY‐0004), [^3^H] PDBu (HY‐18990), and [^3^H] DPB were prepared at the ATCC. The positive control also included reference drugs that included doxorubicin (HY‐15142) and docetaxel (HY‐B0011). The ATCC provided human prostate (PC‐3, DU145) and breast (MCF‐7, MDA‐MB‐231) cells, which were then allowed to grow in normal conditions. The chemicals were first dissolved in dimethyl sulfoxide (DMSO) and then stored at −20°C to make 10‐mM stock solutions. The experiments were conducted with fresh culture media, and the working dilution of the compounds must be prepared immediately before the experiment, with the maximum concentration of DMSO in the solution not exceeding 0.1% (v/v) to prevent cytotoxicity of the solvent.

#### 2.3.3. Ligand Inhibitory Potential

Sal B, EA, [^3^H] DPB, and [^3^H] PDBu have been evaluated for their ability to inhibit DNA lyase, topoisomerase II alpha, and mTOR kinase using enzyme inhibition assays [45, 49]. The compounds were dissolved in DMSO to make 10‐mM stocks, which were then diluted to 0.1–100 *μ*M in order to achieve a dose‐dependent inhibition. The DNA lyase inhibition test was performed using DNA lyase (APE1) and a basic citrate‐containing oligonucleotide substrate. A total of 0.5 *μ*g of dithiothreitol (DTT), 0.5 *μ*g of the DNA substrate, 50 mM Tris‐HCl (pH 7.5), 50 mM NaCl, 10 mM MgCl_2_, and 1 *μ*L of the enzyme made up the reaction mixture. In a 30‐min stop solution of formamide and ethylenediaminetetraacetic acid (EDTA), the incubation was conducted at 37°C. To calculate the percentage of cleavage inhibition in comparison with controls, all samples were denatured and electrophoresed on a 15% denaturing polyacrylamide gel (PAGE). Band intensities were then quantified using a gel documentation system [49]. Supercoiled plasmid DNA (Pbr322) was used as the substrate in a relaxation inhibition test for topoisomerase II alpha. Fifty mM Tris‐HCl, pH 7.9, 120 mM KCl, 10 mM MgCl_2_, 1 mM ATP, 1.0 mM DTT, and varying concentrations of test chemicals made up the assay buffer [49]. The reagents were 0.5 *μ*g of supercoiled plasmid and one unit of human topoisomerase II alpha enzyme. After 30 min at 37°C, the reaction was stopped using sodium dodecyl sulfate (SDS) and EDTA. Proteinase K was optionally added to aid in the enzyme′s digestion. Separations were executed on agarose gel electrophoresis (1% agarose) and stained with ethidium bromide. The supercoiled DNA was subsequently compared with relaxed forms, as a measure of inhibition, with etoposide serving as a positive control. This assay was essential for determining a compound′s affinity for the catalytic TOPRIM domain (also referred to as the etoposide binding pocket) based on the pattern and level of DNA relaxation [56, 57].

The inhibition of mTOR kinase was measured using a colorimetric/fluorescence‐based kinase test kit (ADP‐Glo) that uses a peptide substrate like 4EBP1 and a recombinant human mTOR enzyme. Test chemicals were introduced to the traceable kinase buffer at different doses to conduct reactions. The buffer contained 50 mM HEPES, pH 7.5, 10 mM MgCl_2_, 1 mM DTT, and 100 *μ*M ATP [49]. Following a 1‐h incubation period at 300°C, the kit′s technique for detecting ADP generation was followed. To ensure precise detection, luminescence and fluorescence signals were captured using a microplate reader. The half maximum inhibitory concentration (IC_50_) was calculated using nonlinear regression, and the percentage of inhibition was compared with controls. To confirm the inhibitory assessment, rapamycin was used as a reference standard [58].

## 3. Results and Discussions

### 3.1. Molecular Docking Studies of DNA Lyase

**Figure 1:**
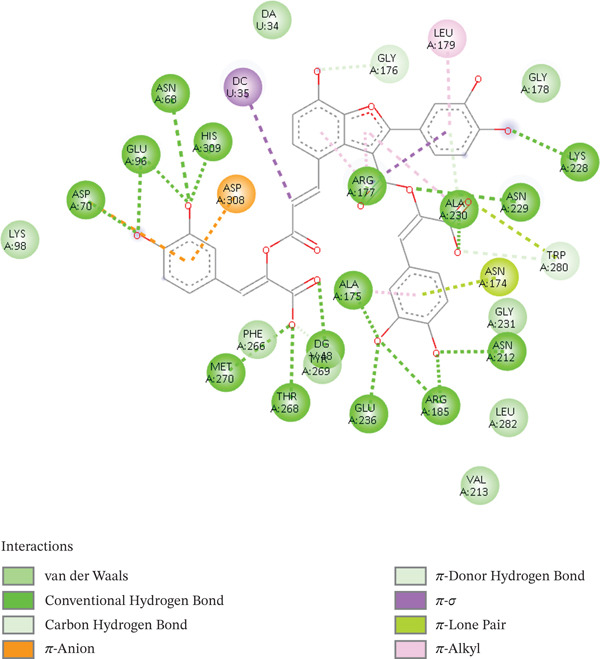
2D interactions of Sal B interacting with DNA lyase.

For visualization of docking scores and poses for each complex, PyMOL and Discovery Studio Visualizer were employed [59, 60]. Sal B formed several hydrogen bonds with important residues ASP210, ASN68, and GLU96, as well as aromatic ring contacts with TYR171, indicating strong binding at the catalytic site, as indicated by its docking score of −9.5 kcal/mol. Consequently, it is known that the polar residues GLU96, ASN68, and ASP10 disrupt enzyme function. The stability and specificity of the receptor–ligand complex are enhanced by the presence of hydrogen bonds in these interactions. Van der Waals forces and hydrogen bonds stabilize the Sal B‐DNA lyase complex, as shown in Figure 1. Sal B exhibits a broad hydrogen‐bonding web interacting with central residues such as GLU236, ASN229, ASN174, and ARG177. Sal B is stabilized in the DNA lyase active site by these polar residues. The aromatic ring of Sal B creates van der Waals and pi–pi bonding interactions in addition to hydrogen bonds with peptide side chains, including TRP280, LEU282, and PHE266. Sal B has an abundance of nonpolar and polar contacts, showing the role of phenolic scaffolds in enzyme inhibition.

It showed promising efficacy against targets related to diabetes, including prostaglandin‐endoperoxide synthase 2 (PTGS2), dipeptidyl peptidase‐4 (DPP4), and glycogen synthase kinase 3 beta (GSK3B). These effects were mediated through aromatic ring interactions, highlighting the versatile pharmacological properties and molecular interactions of Sal B with strong drug‐repurposing potential [61]. In addition, it can be inferred that Sal B may possess anticancer potential due to its stable complex. This reflects prior reports, Guo and Wang [62], that Sal B, who have suggested that Sal B, isolated in the *Salvia* family, might be effective in blocking tumor‐related pathways. The docking scores of common anticancer drugs also lie between −7 and −12 kcal/mol, according to well‐documented literature. VEGF‐2 lipo‐blockers such as axitinib and Smad2/3/MAPK kinase lipo‐blockers are two examples; their respective docking scores are −12 and −10 kcal/mol [63]. Noticeably, Sal B holds a possibly better docking score, making it an excellent DNA lyase inhibitor. The catechol moiety also demonstrated the ability to chelate the catalytic site of the metal ion, showing that the DNA lyase activity was directly interfered with. The long aromatic structure of Sal B enabled its insertion into the DNA binding ridge in a stable manner, promoting its inhibitory effects, reflecting earlier reports on its capacity to interrupt DNA in cells [64]. Figure 1 demonstrates the 2D interactions of Sal B with DNA lyase.

**Figure 2:**
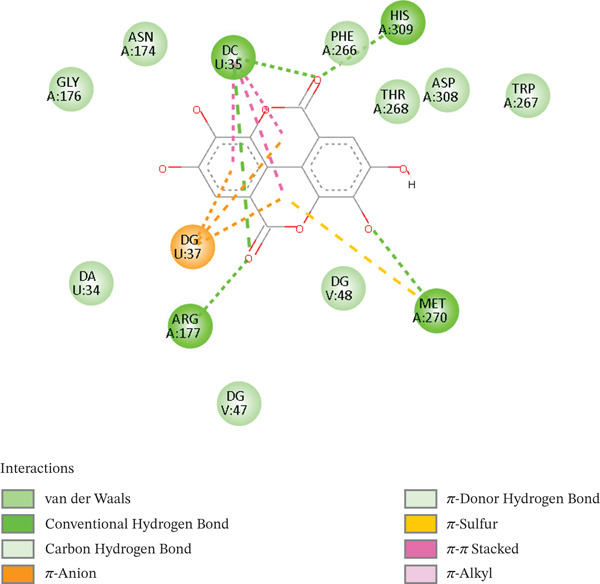
2D interactions of the EA‐DNA lyase complex.

Ellagic acid (EA) is a polyphenolic molecule with a broad pharmacologic profile. EA displays a docking score of −8.7 kcal/mol in this experiment, suggesting that the active site may dock more effectively. Its scoring capability suggests thermodynamically favorable interactions, a feature reflective of a more tightly held and stable complex. Accordingly, complexes with lower (more negative) docking scores are generally more effectively bound to their targets [65]. Consequently, EA enhanced intercalative binding, impeding transcription and replication, whereas the hydroxyl groups interacted with PHE266 and formed stable hydrogen bonds with HIS309 through pi–pi interactions owing to its planar aromatic configuration. Its capacity to form hydrogen bonds with both catalytic residues indicates that it interferes with the recognition and cleavage of AP sites in DNA. It entered the catalytic cleft with little difficulty because of its middle size and polarity. As presented in Figure 2, EA reacts better with nucleobase residues and ARG177, in which the hydrogen bonds are stable. The planar polyaromatic scaffold of EA has a large van der Waals interaction with PHE266, TRP267, and MET270. The reduced number of hydrogen bonds is offset by the nonpolar interactions. EA′s function in filling the limited enzyme pockets is highlighted by the ratios of van der Waals and pi–pi stacking interactions. These findings are consistent with the role that APE1 expression suppression and increased DNA damage in cancer cells play [34]. Thus, EA can inhibit BC and PCa by disrupting normal DNA functions and forming strong hydrogen bonds via the planar stacking of conjugated systems, rendering tumor cells susceptible to treatment. [34]. EA can bind to DNA lyase with comparatively little steric hindrance, making tumors more sensitive to chemotherapy medications, causing DNA damage to accumulate, and causing selective cytotoxicity while protecting normal cells [66]. The experimental research, docking studies, and molecular dynamics are comparable to chemotherapeutics like doxorubicin in that they interfere with DNA base pair transcription and replication. EA is a potential DNA integrity disruptor, a rare genotoxic and phytochemical tumor lead. Figure 2 illustrates 2D interactions between the EA and the DNA lyase protein.

**Figure 3:**
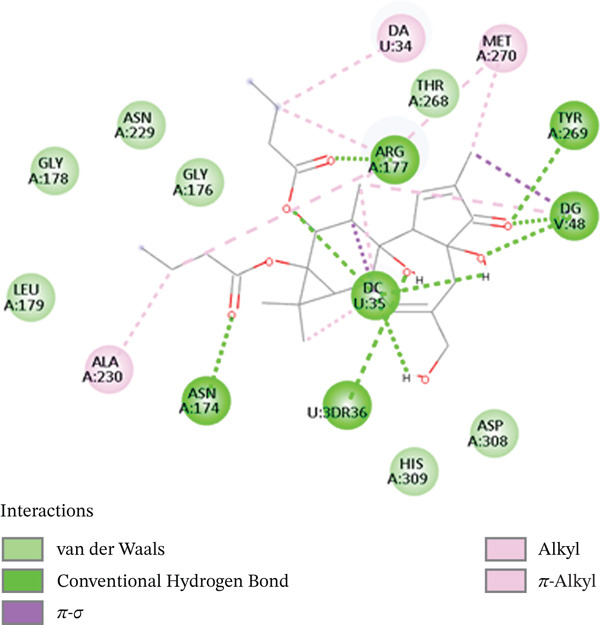
2D interactions of [^3^H] PDBu‐DNA lyase complex.

A moderate docking score value was achieved with [^3^H] PDBu, with a docking score of −7.6 kcal/mol. Binding is generally predicted to occur when the binding energy is negative, with a value of less than −6 kcal/mol. Consequently, the docking scores and interaction profiles of this emerging lead candidate are comparable with those previously reported in the literature, thereby corroborating the reliability of the docking strategy implemented in the present study. The interactions (2D and 3D) between [^3^H] PDBu and DNA lyase are examined and reveal that the protein exhibits hydrophobic and van der Waals interactions, which do not rely solely on metal‐chelating. [^3^H] PDBu forms hydrogen bonding with ARG177, ASN174, and nucleobase residues. Additionally, the phorbol core of this ligand forms van der Waals and hydrophobic bonds with MET270, TRY269, and TRH268, indicating that the hydrophobic interactions stabilize the complex. Without the need for metal coordination, these interactions are essential for keeping the receptor stable in the DNA lyase protein′s binding sites. Notably, the receptor remains highly concentrated in the DNA lyase active site due to its lack of metal‐chelating action. Additionally, the DNA‐binding tunnel′s hydrogen bonds between TRY269 and ARG177 help to disrupt the tumor cell′s DNA repair pathway. This is supported by the presence of His 309, which has a hydrogen bond that could repel the enzymatic action, alters the position of catalytic water. Additionally, [^3^H] PDBu destabilizes the enzyme‐substrate complex by binding to the residues surrounding the region of DNA entry, causing a conformational shift in its target. By increasing DNA damage and interfering with DNA repair processes, the [^3^H] PDBu can improve tumor cells′ susceptibility to therapeutic therapies. By preventing DNA repair, this ligand may potentially aid in the reduction of drug resistance. 2D interactions between [^3^H] PDBu and DNA lyase are shown in Figure 3.

**Figure 4:**
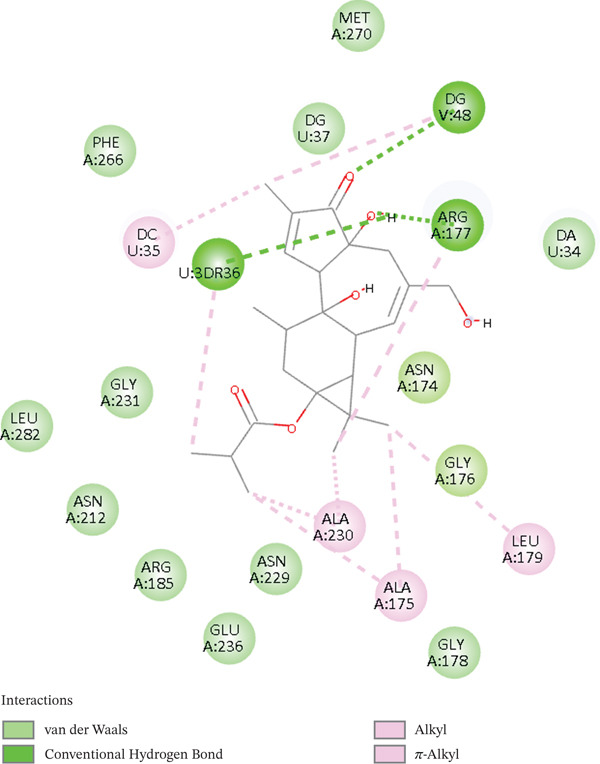
2D structure of [^3^H] DPB‐DNA lyase complex.

Additionally, according to the docking investigations, [^3^H] DPB‐DNA lyase has a docking score of −7.3 kcal/mol, which is somewhat lower than that of its di‐butyrate equivalent. The absence of an oxygen atom at C‐12 resulted in a lower altered conformation, reducing its polar interaction with the enzyme. However, it retained similar hydrophobic and pi–alkyl contacts within the binding pocket, including ARG177, PHE266, and ASN212. At the same time, it does not interact with ASP308 and HIS309, implying a partial obstruction of the catalytic site. Remarkably, [^3^H] DPB ligand stabilizes through van der Waals forces with hydrophobic amino acids, such as LEU179, ALA230, and ASN212. There are also other interactions involved, such as ARG177 and DNA base residues. The inhibitory power of this bioactive molecule is due to the steric bulk and hydrophobic complementation. In all the ligands, hydrogen bonds provide directional anchoring, whereas van der Waals forces are the predominant forces in stabilization. The docking results point to physical obstruction rather than conventional enzymatic inhibition, showing huge prospects of being utilized as a DNA repair inhibitor by targeting its structural vulnerabilities. The contacts, particularly ASN212, PHE266, and ARG177, reside near the DNA‐binding tunnel and can mimic the intercalative effects observed in DNA‐binding drugs. Given its bulk and rigidity, this compound may act through steric hindrance or conformational distortion of APE1, as shown in Figure 4. These results have shown that [^3^H] DPB‐DNA lyase interactions are noncompetitive, broadening the scope of anticancer drug research by introducing molecules that do not need to form polar interactions to enhance their therapeutic efficacy.

### 3.2. Molecular Docking Studies on Topoisomerase II Alpha

Topoisomerase II alpha is recognized as a therapeutic target for anticancer drugs and antimicrobial agents [67]. This anticancer agent works by a mechanism called topoisomerase poisoning, which involves converting the topoisomerase enzyme into a poison ternary complex by covalently trapping it in the active site, thereby forming an intermediate that causes the enzyme to stop functioning. The topoisomerase II alpha complex is a homodimer, in which each protomer is associated with a single DNA molecule. The active site for DNA cleavage and binding includes metal in the TOPRIM domain, a WHD‐bearing tyrosine, and tower domain on both halves of the dimer; isoleucine amino acids play a critical role in the intercalation of the minor groove of DNA, allowing additional contacts. The main amino acids that are responsible for catalysis are TYR805, ASP541, ASP543 coordination with Mg^2+^ ion, GLU416, and ARG804.

**Figure 5:**
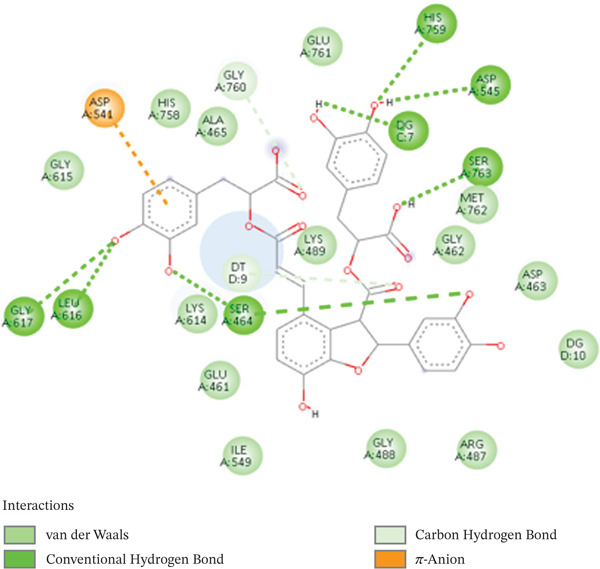
2D interactions of Sal B and topoisomerase II alpha.

Different interaction profiles and docking scores inside the active site of the enzyme are revealed by the docking and interaction profile analysis of substances on topoisomerase II alpha. Sal B engaged important residues TYR805, GLU416, ARG543, and ARG541, displaying the highest docking score of −6.9 kcal/mol. As shown in Figure 5, Sal B interacts with ASP545, ASP541, LYS489, and SER residues in the catalytic and DNA‐binding groove of the enzyme, forming an extensive hydrogen bond network. The polyphenol hydroxyl groups and a few donor and acceptor contacts contribute to the strong anchorage of the ligand on the enzyme. Additionally, van der Waals forces with hydrophobic residues like ALA465, HIS758, and LEU616 support the polyphenolic scaffold′s overall stability. This docking score revealed a thermodynamically stable and strong interaction within the active site. This more negative score corresponds to the predicted docking score achieved through key amino acid residues. Hydrophobic and pi–pi stacking interactions stabilize the orientation of the complex and anchor the receptor on the protein′s active site. The polar residue Gln 416 forms strong hydrogen bonds with hydroxyl groups of Sal B, whereas ARG543 and ARG541 play key roles in electrostatic stabilization by forming hydrogen bonds with carboxylic groups of the receptor. These interactions suggest extensive stabilization through hydrogen bonding and noncovalent pi–electron overlap, especially the aromatic TYR805 near the DNA cleavage/ligation binding site and electrostatic interactions with arginine side chains. Its polyphenolic structure, rich in hydroxyl and carboxyl groups, supports these interactions while offering hydrogen bonding and aromatic stacking. The multifaceted interactions in this complex minimize the likelihood of off‐target effects and enhance potent inhibitions, which are crucial considerations in anticancer drug research. These findings also contribute to the repurposing efforts of Sal B in anti‐inflammatory and cardiovascular applications [64]. The 2D interactions of salvianolic acid and topoisomerase II alpha are shown in Figure 5.

### 3.3. Molecular Docking Studies on Serine–Threonine Kinase mTOR

**Figure 6:**
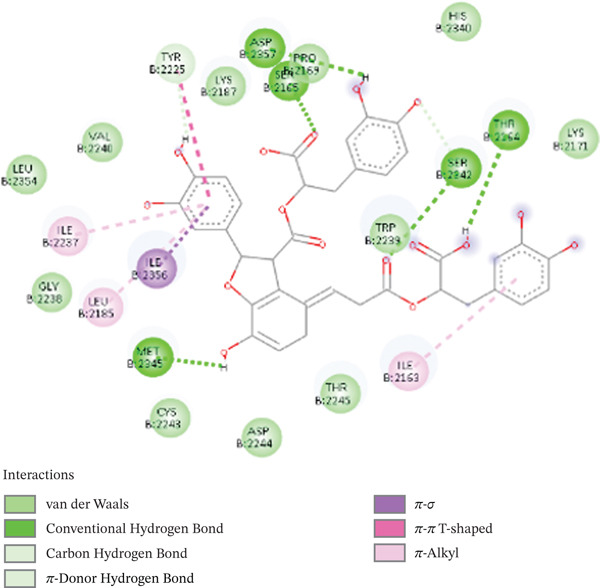
2D structure of Sal B and mTOR interactions.

The docking studies of selected compounds against the mTOR ATP kinase domain reveal a clear trend in docking scores and interaction profiles, which can be rationalized by examining the environment of the receptor and the chemical structures of the compounds. Due to the existence of several hydrogen bond acceptors and donors, such as ASP2338, HIS2430, and ASN2343, which interact with a substantial polar residue network, Sal B has the highest docking score of −7.9 kcal/mol. Because of the polyphenolic structure′s chemical diversity—which contains carboxylic and hydroxyl groups—the complex is thermodynamically stable, according to the docking score. Its multihydroxyl and carboxyl groups on its polyphenolic backbone allow it to participate in several hydrogen bonding contacts and a pi–alkyl reaction with one of the gatekeepers, Ile 2237, a hydrophobic residue. The complex is stabilized by amino acids found in the active cleft, particularly ASN2343, HIS2430, and ASP2338. The anchoring is made possible by the pi–alkyl interactions (Ile 2237), which lengthen the residence duration in the binding position. Its attachment to the ATP kinase pocket, which has hydrophilic and hydrophobic patches, is stabilized by this dual interaction pattern with polar and nonpolar contacts (cf. Figure 6). Sal B exhibits a highly interactional pattern as it forms hydrogen bonds with SER2169, ASN2343, and ASP2575 within the ATP‐binding pocket (Figure 6). Additionally, there are polar interactions that hold the polyphenol scaffold firmly. There are also hydrophobic residues, which include ILE2356, LEU2237, TRY2248, and TRP2239, which form strong van der Waals interactions. Interactions with HIS2430 and ASP2338 are linked to structural flexibility and enzyme activities. These interactions support the potential of the receptor as an anticancer candidate.

Due to its planar, conjugated structure with hydroxyl groups, EA has a stronger ability to generate hydrogen bonds while having a somewhat lower docking score of −7.6 kcal/mol. It interacts hydrophobically with ASP2338, ILE2237, and SER2342 residues and forms hydrogen bonds, particularly with HIS2340. Because it lacks long hydrophobic or flexible moieties, it cannot fully access the hydrophobic portion of the kinase pocket, which results in somewhat less stabilization (cf. Figure 7). Multiple hydroxyl groups make up the planar conjugated aromatic system known as EA, which promotes interactions at the kinase active site. In addition, EA has fewer hydrogen bonds, with the strongest polar contact being VAL2240. However, the van der Waals forces stabilize the planar aromatic systems through residues such as LEU2237, TRP2239, and MET2345. Both van der Waals forces and polar contacts enable EA to fit well within the hydrophobic ATP‐binding channel. EA interferes with the kinase signaling pathways via steric interference. Moreover, hydrophobic contacts: SER2342, ASP2338, and ILE2237, contribute to the ligand stabilization. However, EA lacks long hydrophobic contacts (linkers), and consequently, it lacks enough flexibility to exploit the nearby hydrophobic sites and penetrate deeper into the active site of the enzyme. Sal B exhibits a superior binding profile, interactive versatility, and structural diversity, and EA is limited by polar nature and rigid geometry in the discovery of novel lead candidates.

Figure 73D interactions (a) and (b) 2D interactions of EA‐mTOR complex.

**Figure:**
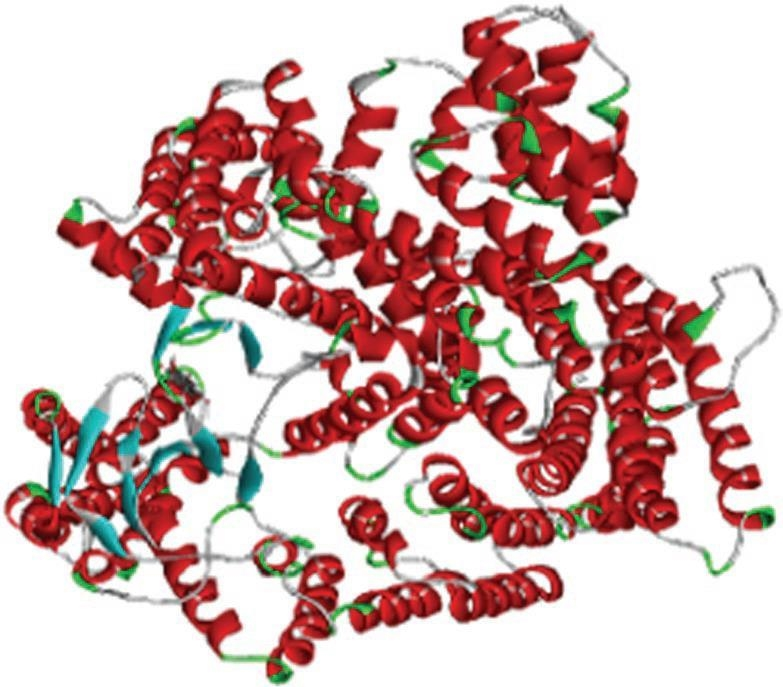
(a)

**Figure:**
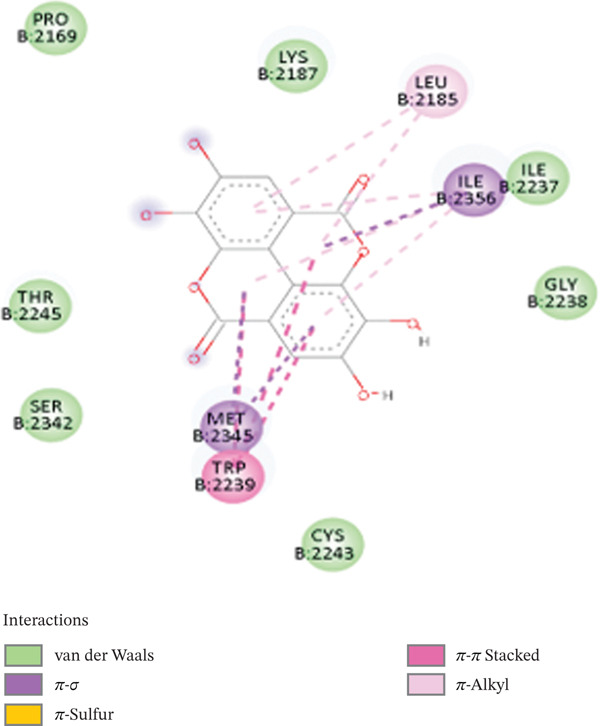
(b)

### 3.4. Docking Validation Protocol

Redocking of co‐crystallized ligands was performed on each protein target into its binding site. Notably, the same binding pose used in the experiment with an RMSD of 1.46 Å was obtained in the active site of APE1. The 2D interaction map of DNA lyase–methoxyamine showed stable hydrogen bonds with ASP308 and ASP70, which are essential catalytic active residues. The crystallographic and re‐docked poses overlap (cf. Figure 8), confirming the accuracy of the docking protocol.

Figure 82D interaction diagram of methoxyamine on active site residues of DNA lyase (a) and (b) superimposition of original pose (yellow) and re‐dock pose (cyan).

**Figure:**
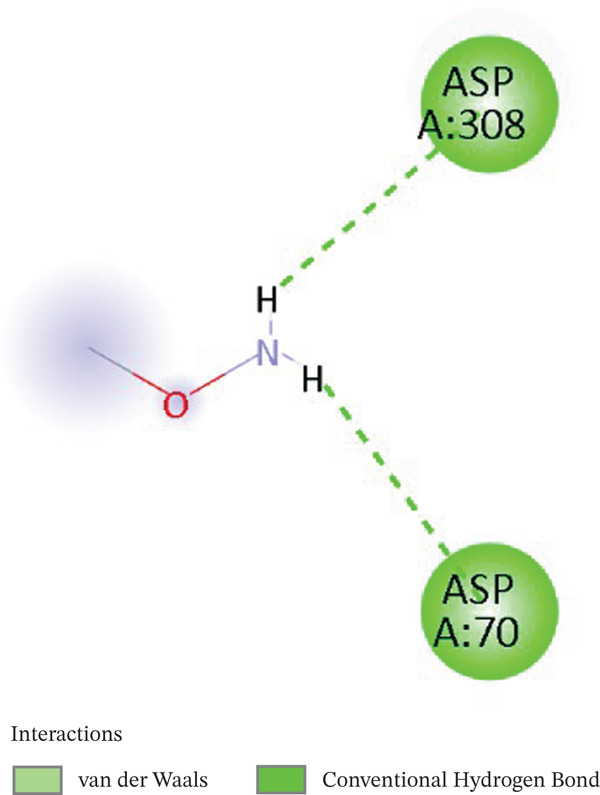
(a)

**Figure:**
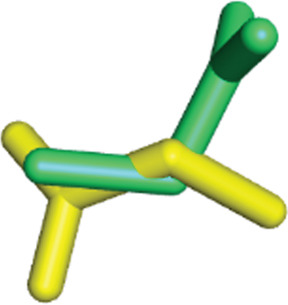
(b)

The docking score of methoxyamine complex was found to be −5.2 kcal//mol, which reflects the findings of other studies that have reported weak‐to‐moderate docking scores of this small AP‐site inhibitor. Better docking scores of −9.1 and −7.4 kcal/mol were shown by Sal B and EA, respectively. Furthermore, methoxyamine had lower docking scores than [^3^H] PDBu and [^3^H] DPB, which were −8.2 and −7.1 kcal/mol, respectively. These higher docking scores could be attributed to large structures that contribute to van der Waals stabilization. Thus, the four bioactive compounds can inhibit APE1 better than the reference drug (methoxyamine), reflecting the findings of previous studies that have shown that phenolic compounds have potent BER pathway inhibitory activity.

Topoisomerase II alpha poison (clinically used drug) was re‐docked successfully into the enzyme pocket with an RMSD of 1.72 Å, relative to its crystallographic orientation. The 2D interaction analysis reveals the presence of hydrogen bonds with ASP541, SER763, and GLU461, as well as hydrophobic stabilization with ARG487 and LEU486, as depicted in Figure 9. The 3D structure shows the consistent positioning of the podophyllotoxin core in both the original and the docked pose. The docking score for etoposide was −9.6 kcal/mol, which is consistent with other scientific reports that have shown that topoisomerase II alpha binds strongly with this standard drug. Whereas [^3^H] DPB showed a docking score of −7.2 kcal/mol, Sal B, EA, and [^3^H] PDBu showed docking scores of −8.8, −8.4, and −7.9 kcal/mol, respectively. These docking score measurements show that these natural compounds can be used as potential inhibitors of topoisomerase II alpha enzyme due to their comparative docking scores with the standard drug (etoposide).

Figure 92D interaction diagram of etoposide on active site residues of topoisomerase alpha II: (a) and (b) superimposition of original pose (orange) and re‐dock pose (pink).

**Figure:**
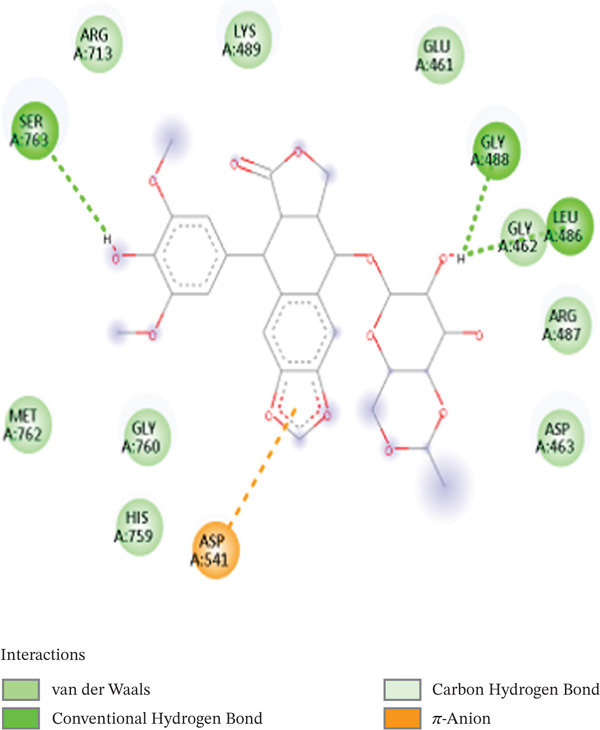
(a)

**Figure:**
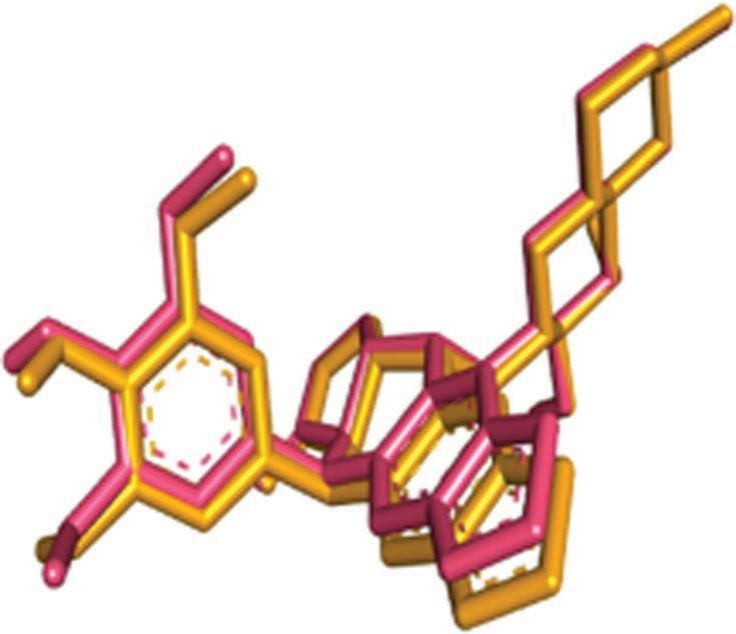
(b)

Rapamycin (clinically approved mTOR inhibitor) was also re‐docked in the kinase binding pocket with an RMSD of 1.55 Å. The docking measurements showed good agreement with experimental findings that have demonstrated the presence of hydrogen bonds with ILE2356, TRP2239, and ASN2237 (c.f Figure 10). The 3D superimposition exhibited close overlap between the macrolide ring, validating the docking grid and scoring measurements, as shown in Figure 10. The docking score for rapamycin of −11.2 kcal/mol reflected its potent allosteric inhibition of mTOR, as observed in biochemical and crystallographic studies. Remarkably, the docking scores for Sal B (−9.0 kcal/mol), EA (−7.6 kcal/mol), [^3^H] PDBu (−8.7 kcal/mol), and [^3^H] DPB (−7.1 kcal/mol) are lower than that of rapamycin. Although these natural compounds cannot outcompete the standard drug (rapamycin), they can be used as complementary inhibitors to stabilize the ATP pocket via van der Waals interactions. Re‐docking co‐crystallized ligands with an RMSD of 2.0 Å or lower validated the docking protocol employed in this study. Also, the standard drugs (methoxyamine, etoposide, and rapamycin) exhibited docking scores that matched their reported pharmacological profiles, and the natural products (diterpene esters and polyphenols) have shown their potential as anticancer agents, as summarized in Table 1.

Figure 102D interaction diagram of etoposide on active site residues of topoisomerase alpha II (a) and (b) superimposition of original pose (orange) and re‐dock pose (pink).

**Figure:**
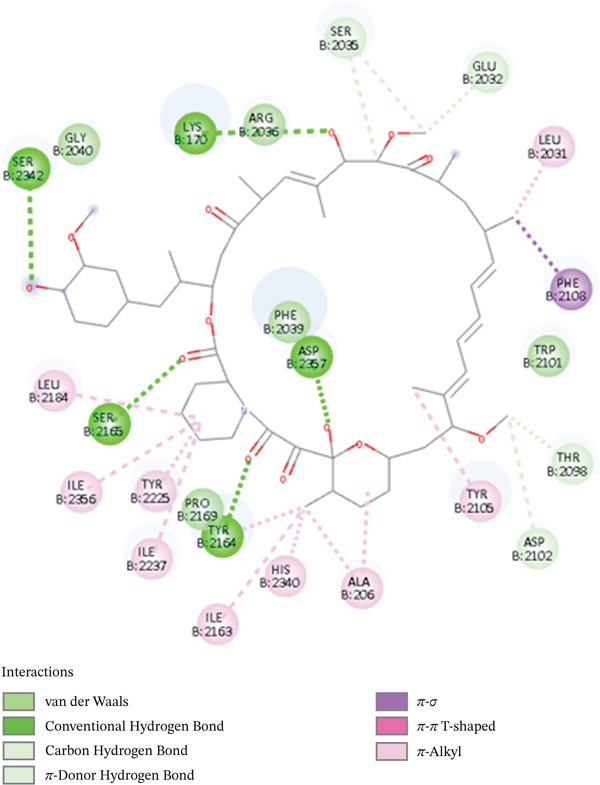
(a)

**Figure:**
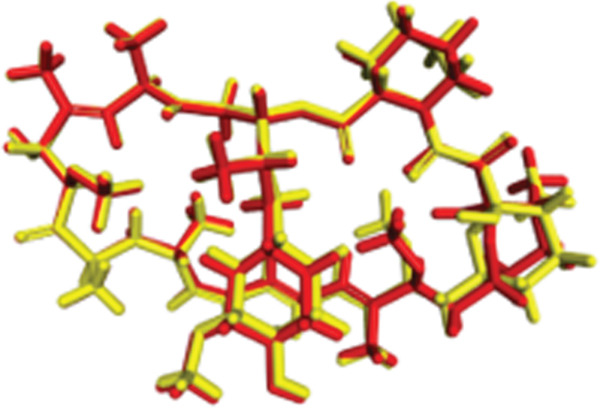
(b)

**Table 1:** Docking scores of ligands in comparison with reference drugs.

Target enzyme	Reference drug (docking score) in kcal/mol	Sal B	EA	[^3^H] PDBu	[^3^H] DPB
DNA lyase (APE1)	Methoxyamine (−5.2)	−9.1	−7.4	−8.2	−7.1
Topoisomerase II*α*	Etoposide (−9.6)	−8.8	−7.9	−8.4	−7.2
mTOR kinase	Rapamycin (−11.2)	−9.0	−7.6	−8.7	−7.1

## 4. Chemical Descriptors

The molecular orbitals have been analyzed in detail using DFT to gain insights into the electronic configuration of the molecules under study [68]. The molecular stability and reactivity of molecules can be predicted using the highest occupied molecular orbital (HOMO) and the lowest unoccupied molecular orbital (LUMO). A low *Δ*E_Gap_ is synonymous with high reactivity and polarity, negatively influencing the molecular efficiency in biological interaction. Conversely, a lower *Δ*E_Gap_ indicates less reactivity and greater stability, reflecting the propensity for molecular softness. Table 2 presents the chemical descriptor data of the compounds studied.

**Table 2:** Chemical descriptor data.

Ligand	Dipole moment (Debye)	HOMO (eV)	LUMO (eV)	Energy gap (*Δ* *E*_*G* *a* *p*_)	*I*(eV)	*A*(eV)	*χ*(eV)	*μ*(eV)	*η*(eV)	*σ*(eV)	*ω*(eV)
Sal B	27.52	−4.99	−4.44	0.55	5.00	4.45	4.725	−4.725	0.2745	1.818	40.56
EA	4.022	−5.93	−1.80	4.12	5.928	1.801	3.864	−3.864	2.603	0.485	3.602
[^3^H] PDBu	3.06403	−6.13	−1.12	5.01	6.126	1.122	3.624	−3.624	2.502	2.625	0.399
[^3^H] DPB	5.0786	−6.18	−1.21	4.97	6.179	1.207	3.693	−3.693	2.486	2.742	0.402

As depicted in Table 2, Sal B has the largest dipole moment, indicating a highly polarized charge distribution that enables electrostatic interactions with polar residues in the binding pocket. Such high polarity is specifically desirable when interacting with charged or polar residues in the catalytic cores of drug targets. Conversely, EA has a dipole moment of 4.022 Debye, whereas phorbol esters [^3^H] PDBu and [^3^H] DPB have 3.064 and 5.078 Debye, respectively. Further, Sal B has a small HOMO‐LUMO gap (*Δ*E_Gap_ = 0.55 eV), increasing its propensity to be more chemically reactive and to exchange electrons. However, this *Δ*E_Gap_ shows that Sal B has lower kinetic stability. The electronegativity (*χ* = 4.725 eV) and the chemical potential (*μ* = −4.725 eV) also indicate that Sal B is an electron tightly accepting functional group, which complements the electron‐donating nature of the amino acid residues, lysine, arginine, and histidine. It is fairly soft and highly electrophilic (*ω* = 40.56 eV), and it can readily accept electron density transferred by nucleophilic amino acid side chains, stabilizing the ligand in the binding pocket due to favorable electrostatic and orbital interaction.

EA has a fairly high electronegativity (*χ* = 3.864 eV) and a substantially higher HOMO‐LUMO gap (4.124 eV). Therefore, it is less reactive as well as less capable of making charge–transfer interactions with the protein. Nevertheless, it was moderately electrophilic (*ω* = 3.602 eV) with a favorable ionization potential (I = 5.928 eV) and so stronger but not as strong interactions as Sal B were possible. The lesser softness (*σ* = 0.485 eV) value of the EA suggests a stiffer electron cloud. [^3^H] PDBu and [^3^H] DPB have higher HOMO‐LUMO gaps (5.007 and 4.973 eV, respectively), which were indicative of their low reactivity and inability to form strong polar or charge–transfer interactions with protein targets. However, these esters have higher kinetic stability, which may be beneficial for participating in chemical reactions that exert therapeutic effects. Their therapeutic application may be impeded by their less electronegative nature and lower *μ* as compared with Sal B and EA, indicating a lower possibility of taking electrons away from donor residues. In addition, the two phorbol derivatives have low *ω* (0.399 eV–0.402 eV) and *σ* (2.625 eV‐2.742 eV), suggesting weak and transient binding interactions.

### 4.1. Molecular Dynamics Simulation

The accuracy of the docking protocol and high stabilization at the simulation platform, as measured by the number of hydrogens and RMSD, were assessed in this study using molecular dynamics simulations on a protein–ligand complex [69]. Protein structural stability has been evaluated using the RMSD measurements. The average RMSD values for Sal B and the EA complex in this investigation were found to be 0.366 and 0.32 nm, respectively. Protein–ligand complex conformational stability is indicated by RMSD values smaller than 0.3 nm, according to earlier research. Noticeably, the RMSD varied in the first 50 ns of the molecular dynamics trajectories in the binding of Sal B and EA in the target proteins′ active pockets in both complexes (RMSD values < 0.3 nm). This behavior is explained by the ligands’ particular alignment with the protein′s active pocket and their great kinetic stability [70]. However, as the simulation progressed, the system stabilized, albeit with a few slight RMSD variations. Following ligand binding, the complexes′ RMSD plots showed very little structural variation once they had reached equilibrium and steady state. The directionality and stability of protein–ligand interactions were evaluated by analyzing the hydrogen bond network on Sal B and EA complexes over a 200‐ns simulated period. There is a correlation between the stability and structural integrity of the protein–ligand complex and the quantity of hydrogen bonds. Noticeably, there were no discernible variations in the overall number of hydrogen bonds formed by the two complexes throughout a 200‐ns period. It was determined that Sal B and EA had an average of approximately three hydrogen bonds. Figure 11 displays the RMSD plots and the quantity of hydrogen bonds in the Sal B and EA ligands. According to the results, the docked complexes were stable, and the complexes′ intramolecular hydrogen bonding contacts were strong and long lasting [68]. These findings support the accuracy and dependability of docking predictions by validating the interaction patterns seen during molecular docking simulation [60, 71].

Figure 11The root mean square deviation for (a) Sal B, (b) EA, and the number of hydrogen bonds for (c) Sal B and (d) EA.

**Figure:**
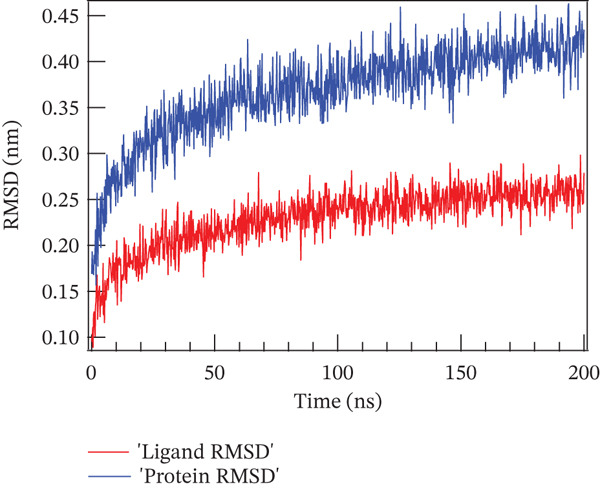
(a)

**Figure:**
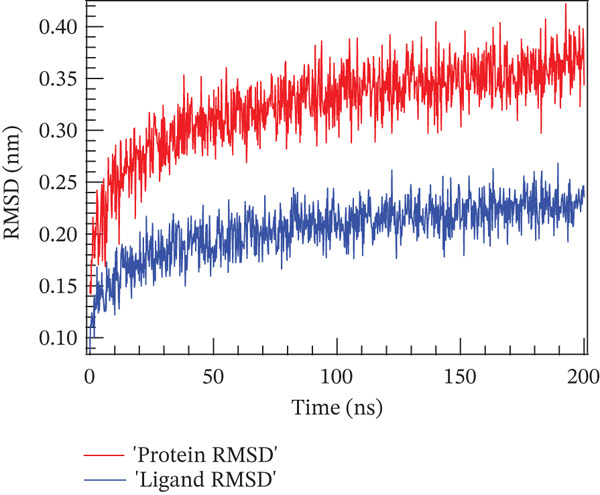
(b)

**Figure:**
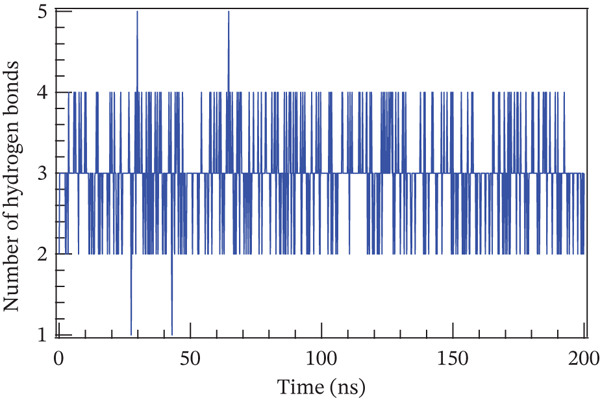
(c)

**Figure:**
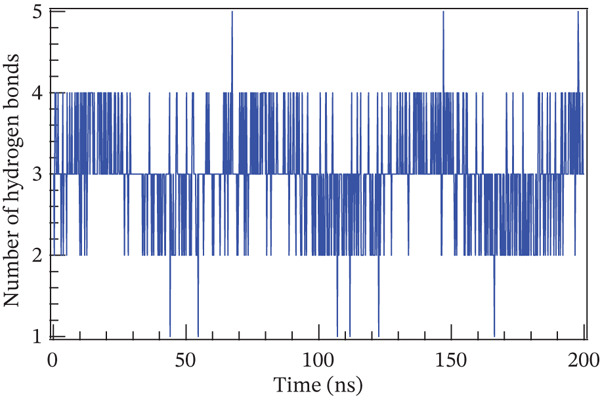
(d)

### 4.2. In Vitro Enzyme Inhibition Assay

**Figure 12:**
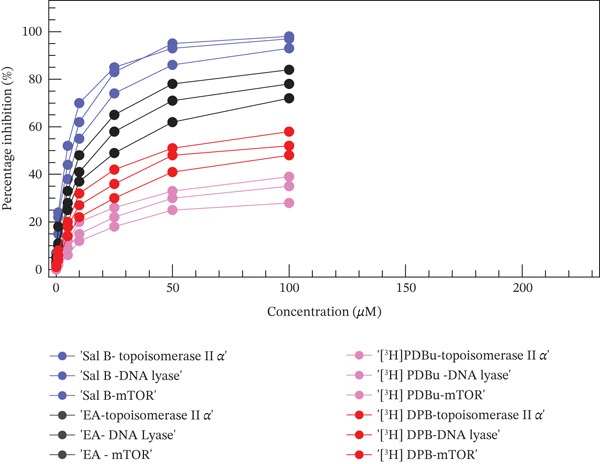
Percentage inhibition of ligands on selected protein targets.

Upon plasmid‐based cleavage assay, the inhibition of DNA lyase was monitored by the prevention of nicked DNA formation. Sal B showed potent inhibition with an IC_50_ of 18.2 *μ*M, reducing enzyme activity by 80% at 50 *μ*M. EA exhibited moderate inhibition with an IC_50_ of 28.7 *μ*M by 60% inhibition, whereas [^3^H] PDBu was ineffective primarily in the assay, showing < 20% inhibition even at a higher concentration of 100 *μ*M, and [^3^H] DPB showed marginally higher inhibition with an estimated IC_50_ of 60 *μ*M with 35% inhibition at 100 *μ*M. Topoisomerase II alpha activity was assessed using a decatenation assay involving kinetoplast DNA substrates. Accordingly, the loss of decantation quantifies inhibition. Salvianolic acid showed strong dose‐dependent inhibition, with complete inhibition of 95% observed at 50 *μ*M and an IC_50_ of 14.8 *μ*M. Even at a lower concentration of 10 *μ*M, it still inhibited over 50% of enzyme activity. EA demonstrated moderate inhibition, with an IC_50_ of 22.6 *μ*M, and the inhibition plateaued around 70% even at 100 *μ*M. In this study, [^3^H] PDBu exhibited poor inhibition, achieving only 25%–30% activity suppression even at 100 *μ*M with an estimated IC_50_ > 70 *μ*M, whereas [^3^H] DPB showed slightly better inhibition but still poor, achieving 45%–50% inhibition at 100 *μ*M and an IC_50_ of 52 *μ*M. An ATP‐competitive fluorescence‐based mTOR kinase assay was used to determine the inhibitory effect of test compounds. Sal B showed high potency with an IC_50_ of 11.6 *μ*M, nearly comparable with that of the standard inhibitor, rapamycin (IC_50_ of 5.2 *μ*M). EA showed moderate inhibition with an IC_50_ of 21.9 *μ*M. The inhibitory activity of [^3^H] PDBu was negligible, with an IC_50_ of >75 *μ*M, whereas [^3^H] DPB showed slightly better but still poor IC_50_ of 58 *μ*M. The inhibitory potential of the selected ligands is summarized in Tables 3 and 4, and Figure 12.

**Table 3:** Ligands′ inhibitory potential.

Compound	Topo II*α* IC50 (*μ*M)	DNA lyase IC50 (*μ*M)	MTOR IC50 (*μ*M)
Sal B	14.8	18.2	11.6
EA	22.6	28.7	21.9
[^3^H] DPB	52	60	58
[^3^H] PDBu	> 70	> 90	> 75
Etoposide (topo II)	5.2	—	—
Methoxyamine (lyase)	—	8.7	—
Rapamycin (mTOR)	—	—	5.2

**Table 4:** Percentage inhibition of compounds at varying concentrations.

Concentration of compounds (*μ*M)	Sal B			EA			[^3^H] PDBu			[^3^H] DPB		
	Topo II *α*	DNA lyase	mTOR	Topo II*α*	DNA lyase	mTOR	Topo II*α*	DNA lyase	mTOR	Topo II*α*	DNA lyase	mTOR
0.1	5%	3%	7%	3%	2%	5%	0%	0%	1%	1%	1%	2%
1	22%	15%	24%	11%	10%	18%	3%	2%	5%	6%	4%	8%
5	44%	38%	52%	28%	25%	33%	9%	6%	12%	18%	14%	20%
10	62%	55%	70%	41%	37%	48%	15%	12%	20%	27%	22%	32%
25	83%	74%	85%	58%	49%	65%	22%	18%	26%	36%	30%	42%
50	95%	86%	93%	71%	62%	78%	30%	25%	33%	48%	41%	51%
100	98%	93%	97%	78%	72%	84%	35%	28%	39%	52%	48%	58%

## 5. Conclusions

Molecular docking studies revealed that Sal B exhibited the highest docking score with all enzyme catalytic residues through key hydrogen bonding, followed by EA, which showed moderate binding affinity. However, [^3^H] PDBu and [^3^H] DPB displayed significantly weaker binding on all enzymes except DNA lyase. Biochemical inhibition analysis supported the computational findings that Sal B is a potential inhibitor of all the targets in anticancer drug development. Accordingly, the IC_50_ values showed good agreement with in silico studies that Sal B is the most promising inhibitor across all targets, whereas EA was moderately effective, and phorbol compounds were less effective, with only promising inhibition of DNA lyase observed, compared with standards such as methoxyamine, etoposide, and rapamycin. The docking protocol was validated by low RMSD values of less than or equal to 2 Å, and the molecular dynamics simulations confirmed the stable conformational integrity and hydrogen bonding that underscored the potential of EA as an anticancer agent. Accordingly, the coupled approach of integrating molecular dynamics, molecular docking, redocking validation, and in vitro assay provides a robust platform to benchmark *Sapium* metabolites. In addition, this comparative framework has highlighted the limited but target‐specific activity of phorbol esters. Ultimately, the potential preventive and therapeutic effects of these bioactive compounds must be confirmed through precise clinical trials and in vivo studies.

## Author Contributions

**R.B.O.O.**: original draft, method development, writing, and editing; **J.K.K.**: conceptualization, editing, and supervision; **S.M.N.:** visualization, editing, and supervision; **U.R.**: writing—original draft, methodology, visualization results, and software; **M.W.A.**: writing—md simulation, visualization, software, methodology, and formal analysis; **U.N.**: writing, conceptualization, methodology, visualization, and software.

## Funding

No funding was received for this manuscript.

## Disclosure

All authors have read and approved the manuscript.

## Ethics Statement

The authors have nothing to report.

## Conflicts of Interest

The authors declare no conflicts of interest.

## Data Availability

The data that support the findings of this study are available from the corresponding author upon reasonable request.
